# Health Care–Related Savings Accounts, Health Care Expenditures, and Tax Expenditures

**DOI:** 10.1001/jamahealthforum.2024.2896

**Published:** 2024-09-20

**Authors:** Dong Ding, Sherry Glied

**Affiliations:** 1 Robert F. Wagner Graduate School of Public Service, New York University, New York

## Abstract

**Importance:**

Approximately 30% of US families with employer-sponsored health insurance, disproportionately drawn from high-income groups, benefit from flexible spending accounts (FSAs) or health savings accounts (HSAs). The combined association through both out-of-pocket spending and premiums of these tax-favored accounts with health care expenditures and tax expenditures remain uncertain.

**Objective:**

To compare the health care and health-related tax expenditures among families holding FSAs, HSAs, or neither type of account.

**Design, Setting, and Participants:**

This cross-sectional study used family-level data from the Medical Expenditure Panel Survey from January 1, 2011, to December 31, 2019, and conducted regression models, controlling for demographic and socioeconomic characteristics, chronic conditions, prior health care expenditures, and marginal tax rates to analyze how holding tax-favored accounts is associated with families’ health care spending and tax expenditures. The sample was restricted to families included in the survey for 2 years, with no members 65 years or older, and with at least 1 policyholder covered (only) by full-year employer-sponsored insurance. Data were analyzed from December 1, 2023, to April 30, 2024.

**Exposures:**

Holding FSAs or HSAs.

**Main Outcomes and Measures:**

Out-of-pocket and insurance-paid health expenditures overall and by service were measured. Health-related tax expenditures were based on tax-excluded insurance premiums and tax-sheltered out-of-pocket expenses.

**Results:**

Of the 17 038 families included in the study sample, 2628 held FSAs (weighted 17%) and 1845 (weighted 13%) held HSAs. In regression-adjusted models, families with FSAs spent a mean of 20% or $2033 (95% CI, $789-$3276) more on health care annually than non–account holding families, largely due to increased insurer-paid expenses. Families with HSAs spent a mean of 44% or $697 (95% CI, $521-$873) more on out-of-pocket expenditures and had insignificantly higher insurance-paid expenditures than families without accounts, resulting in overall expenditures comparable to those of non–account holders. The additional tax expenditures associated with FSAs were a mean of $1306 (95% CI, $536-$2076) annually per family. Both types of funds were associated with significant increases in tax expenditures from additional office-based visits ($445 [95% CI, $244-$645] for FSAs and $174 [95% CI, $11-$336] for HSAs), outpatient visits ($330 [95% CI, $132-$528] for FSAs and $250 [95% CI, $15-$485] for HSAs), dental visits ($180 [95% CI, $126-$233] for FSAs and $165 [95% CI, $104-$226] for HSAs), and vision care ($36 [95% CI, $28-$45] for FSAs and $52 [95% CI, $40-$64] for HSAs).

**Conclusions and Relevance:**

Participation in FSAs is associated with higher health care expenditures and tax expenditures, while HSAs are not associated with reduced expenditures. Tax policy could be better targeted to enhance insurance coverage and health care accessibility.

## Key Points

**Question:**

To what extent is holding flexible spending accounts (FSAs) or health saving accounts (HSAs) associated with health care expenditures and health-related tax expenditures among US families with employer-sponsored health insurance?

**Findings:**

In this cross-sectional study of 17 038 families, those with FSAs spent 20% more annually on health care, primarily due to higher insurer-paid expenses, while families with HSAs had overall spending comparable to those without tax-favored accounts. The annual tax expenditure associated with increased FSA spending was a mean of $1306 per family holding this type of account.

**Meaning:**

These findings suggest that tax-favored health spending accounts, which increase (FSA) or fail to reduce (HSA) spending and primarily benefit high-income families, reduce the equity of the health care system and do not improve its efficiency.

## Teaser

This cross-sectional study compares out-of-pocket and insurance-paid health care expenditures and related tax expenditures among families covered by flexible savings accounts, health savings accounts, or neither.

## Introduction

Approximately 22% of US residents aged 19 to 64 years with employer-sponsored health insurance (ESI) currently hold a tax-favored account in tandem with that insurance, most commonly, a flexible spending account (FSA) or a health savings account (HSA), though other types of tax-favored health accounts also exist. The FSA provisions, which allow employees to set aside a portion of their pretax income to cover qualified medical expenses, were legislated as part of the Revenue Act of 1978. Congress created tax-favored HSAs in 2003 through the Medicare Prescription Drug, Improvement, and Modernization Act. The HSA provisions also allow employees to save pretax income to cover qualified medical expenses and, unlike FSAs, HSA balances roll over from year to year. However, only employees enrolled in high-deductible health plans (HDHPs) may contribute to HSAs. Funds in both types of accounts may be used to cover eligible medical expenses of the fundholder and their eligible spouses and dependents. There is growing interest in expanding access to such accounts.^1,2^

Economists have long noted that the tax-favored status of ESI encourages people to purchase more generous plans through their employers than they otherwise would,^3,4^ which may contribute to inefficient use of health care services.^5^ Tax-favored accounts supplement the existing tax benefits and could exacerbate or diminish the inefficient effects of the tax treatment of ESI. Conditional on a given level of deductibles and coinsurance, FSAs and HSAs reduce the after-tax effective rate of cost-sharing.^6^ Reducing this effective cost-sharing rate may lead to higher overall health care expenditures, potentially raising premiums.^7^ Tax-favored accounts may also encourage individuals to purchase tax-favored services that are not covered by their insurance, such as dental care and eyeglasses. This is particularly likely for FSAs, because the “use-it-or-lose-it” rule may encourage people to spend money left in their accounts at the end of each year on items they may not actually need,^8,9^ though in practice, HSA holders also spend most (85%) of the funds they deposit and few carry over and invest balances.^7,10^

Alternatively, extending tax benefits to out-of-pocket payments could offset the perverse effects of the favorable tax treatment of ESI. Shielding both premiums and out-of-pocket payments from taxes could encourage consumers to choose less generous plans, with higher coinsurance and deductibles, reducing insurance premiums. This generosity-reducing effect is more evident in the case of HSAs, where tax benefits are conditioned on the purchase of an HDHP, but the availability of FSAs may likewise make plans with higher cost-sharing more attractive to employers and their employees.^11,12,13,14,15^ Higher cost-sharing induced by new tax benefits could reduce overall health care expenditures.^16,17,18^

The Joint Committee on Taxation^19^ reported that, in 2022, employer contributions for ESI premiums accounted for $187.4 billion in federal tax expenditures—that is, taxes that would have been collected had compensation been paid in wages—and HSAs accounted for $11 billion in federal tax expenditures. However, the Joint Committee on Taxation estimate is incomplete because it does not take into account the potential effects of savings accounts on premiums. Tax expenditures associated with FSAs and HSAs might be misestimated under 3 scenarios. First, tax expenditures might be underestimated if sheltering out-of-pocket spending from taxation induces greater overall health care spending. In this scenario, accounts would directly generate higher tax expenditures through newly sheltered out-of-pocket costs and indirectly generate higher tax expenditures through higher premiums. Second, tax expenditures could be overestimated if participation in these plans induces consumers to choose plans with higher cost-sharing, leading to less spending and substantially lower tax-favored premiums. Third, tax expenditures could be underestimated even if tax-favored accounts lead consumers to choose plans with lower premiums, but the premium reduction effect is less than the tax-favored out-of-pocket spending effect.

## Methods

In this cross-sectional study, we used the Medical Expenditure Panel Survey (MEPS) data at the family level from January 1, 2011, to December 31, 2019, to compare health care expenditures (including out-of-pocket and insurance-paid expenditures on office-based, outpatient, emergency department, and dental visits; inpatient stays; prescriptions; and vision care) and related tax expenditures among families covered by FSAs, HSAs, or neither. We did not include data after 2020 because of the confounding effects of the COVID-19 pandemic. We analyzed these data between December 1, 2023, to April 30, 2024. As the study involved secondary analysis of publicly available data (the MEPS), it did not require institutional review board approval according to New York University guidelines. This study meets the Strengthening the Reporting of Observational Studies in Epidemiology (STROBE) reporting guidelines for cross-sectional studies.

### Data Sources and Study Design

The MEPS is a nationally representative survey of the US civilian noninstitutionalized population. We restricted the sample to families who stayed in the survey for 2 years, with no members 65 years or older in either year, and at least 1 policyholder covered (only) by full-year ESI. Our final analytic sample includes 17 038 families (after weighting, this represents about 24.5 million families per year). For families with more than 1 policyholder (12% of the sample), we assign a primary holder (the FSA or HSA holder or the primary tax filer if indicated; if not, the adult with the highest income; if a tie, the reference person).

### Variables

#### FSAs and HSAs Holding and Pretax Out-of-Pocket Spending

Information on HSA holding is available in the MEPS Person Round Plan files, while FSA holding information and self-reported contributions are in the MEPS Full-Year Consolidated Data Files (other types of savings accounts are not included in the MEPS Household Component data). We defined families holding FSAs or HSAs as those with at least 1 account holder covered solely by full-year ESI.

We assumed that the total out-of-pocket spending for each account-holding family is covered by pretax dollars unless it exceeds a threshold. For families with FSAs, the threshold is the lesser of the family’s self-reported FSA contribution or the FSA limit. For families with HSA, we used data from an annual report by the Employee Benefit Research Institute (2011-2019)^10^ on account balances (initial balance plus annual contribution) matched to the account holders’ age (including the approximately 50% of holders in the Employee Benefit Research Institute database with no contributions). If a family’s total out-of-pocket spending exceeded the pretax spending threshold, only the threshold amount is considered pretax. For families holding both FSAs and HSAs (4% of the sample), we deferred to the higher pretax out-of-pocket spending and recoded the family accordingly.

#### Quasi-Premiums

The MEPS does not provide direct information on total (employer plus employee) insurance premiums. We therefore constructed simulated quasi-premiums based on each family’s actual insurance-paid expenditures. We adjusted the MEPS insurance-paid expenditure data (excluding dental and vision care) for underreporting of insurance payments, using a 29% inflation factor suggested by Zuvekas,^20^ so that mean insurance-paid expenditures in our sample matched insurer-reported amounts.^21^ We then added a loading cost of 16%, as suggested by Karaca-Mandic et al.^22^ Our resulting quasi-premium estimates are close to the corresponding premiums reported by the MEPS Insurance Component^23^ for single individuals and families of 2, but about 20% below MEPS Insurance Component measurements for larger families (a conservative assumption). We assume that both employer and employee premiums are paid with pretax dollars.

#### Marginal Tax Rate and Health-Related Tax Expenditure

We estimated each family’s marginal tax rate using the National Bureau of Economic Research TAXSIM model.^24^ The public-use MEPS data include region but not state identifiers. We assigned each family to a state within its region based on the state’s share of the region’s population from the US Census Bureau.^25^ We calculated health-related tax expenditures from the tax exclusion of quasi-premiums and from out-of-pocket spending covered by pretax funds by applying marginal tax rates to these quantities. We applied federal and state marginal income tax rates, and the Federal Insurance Contributions Act (FICA) marginal tax rate^26^ to calculate federal, state, and FICA tax expenditures.

### Statistical Analysis

We drew our primary outcomes—health care expenditures and health-related tax expenditures—and FSA and HSA holding status from the second year of survey participation. All expenditures were inflated to 2023 US dollars using the Consumer Price Index. We first described the characteristics (in the second year of participation) of families in our analytical sample holding FSAs, HSAs, or neither. Next, to compare the unadjusted means of health care expenditures among the 3 groups of families, we estimated a 2-part health expenditure model, with probit model in the first part and generalized linear model with log transformation in the second part, as this model adjusts for the high frequency of zero expenditures and the skewed distribution of positive expenditures,^27^ initially including only fixed effects for the year.

In our main 2-part model, we controlled for factors previously identified as affecting FSA and HSA enrollment: health care expenditures, marginal tax rates, and the number of family members who had MEPS-reported chronic conditions drawn from the first year of the survey.^28,29,30^ We also controlled for factors that have previously been shown to affect health expenditures and that are correlated with holding FSAs or HSAs. These factors included family characteristics (number of male and female adults and children, region, income, number of ESI beneficiaries, and number of individuals older than 45 years) and primary policyholder attributes (age, sex, race and ethnicity, educational attainment, employment, and marital status) reported in the second year of survey participation.

We did not control for HSA or FSA holding status in the first year of survey participation because the carryover of funds makes it challenging to attribute tax expenditures. Our results are not sensitive to this exclusion. In estimating tax expenditures on pretax out-of-pocket spending, we restricted the sample to families who hold FSAs or HSAs, as only those with accounts can have pretax out-of-pocket expenditures. We reported estimates for total, out-of-pocket, and insurance-paid health care expenditures; for each tax component (federal, state, and FICA); and by type of service. In robustness analyses, we considered alternative functional form specifications and estimated the potential effects of omitted variables. Two-sided *P* < .05 indicated statistical significance, and analyses used Stata, version 18.0 (StataCorp LLC).

## Results

### Characteristics of Families Who Hold FSAs and HSAs

From 2012 to 2019, 17 038 families were included in the study population, including 2628 with FSAs and 1845 with HSAs. A weighted 17% of full-year ESI-covered families had an FSA, while the weighted proportion of families with an HSA increased from 10% to 17% (mean, 13%). More than two-thirds of families with FSAs or HSAs (75% with FSAs and 68% with HSAs) had household incomes exceeding 400% of the federal poverty level (Table). Consistent with prior studies, 3 of 5 account holders attained a bachelor’s degree or higher, and a weighted 7% were Asian, 8% were Black, 7% were Hispanic, 77% were White, and 2% were multiracial or other race (including American Indian or Alaska Native and Native Hawaiian or other Pacific Islander).^31,32^

**Table.** Family and Primary Policyholder Characteristics

Characteristic	Account held^a^		
	FSA (n = 2628)	HSA (n = 1845)	Neither (n = 12 565)
**Family **			
No. per household, mean (SD)			
Adults	1.86 (0.63)	1.78 (0.60)	1.70 (0.70)
Children	0.79 (1.00)	0.75 (0.99)	0.62 (1.01)
ESI-covered individuals	2.56 (1.28)	2.37 (1.27)	2.12 (1.33)
ESI holders	1.14 (0.34)	1.17 (0.36)	1.12 (0.36)
FSA holders	1.15 (0.38)	0.00 (0.00)	0.00 (0.00)
HSA holders	0.00 (0.00)	1.03 (0.17)	0.00 (0.00)
Type of family			
1 Person	0.21 (0.19-0.23)	0.27 (0.24-0.30)	0.29 (0.28-0.30)
2 People	0.25 (0.23-0.27)	0.25 (0.23-0.27)	0.25 (0.23-0.27)
>2 People	0.54 (0.51-0.56)	0.45 (0.45-0.51)	0.46 (0.45-0.47)
Region			
Northeast	0.18 (0.15-0.21)	0.16 (0.14-0.18)	0.20 (0.19-0.21)
Midwest	0.25 (0.22-0.28)	0.30 (0.27-0.33)	0.21 (0.19-0.22)
South	0.36 (0.33-0.40)	0.33 (0.30-0.36)	0.36 (0.34-0.38)
West	0.21 (0.19-0.23)	0.21 (0.18-0.24)	0.23 (0.21-0.25)
Family income, mean (SD), $	152 962 (84 734)	141 528 (81 119)	117 620 (80 143)
**Primary policyholder **			
No. male, mean (SD)	0.50 (0.47)	0.57 (0.46)	0.57 (0.51)
No. married, mean (SD)	0.70 (0.43)	0.65 (0.44)	0.56 (0.51)
Age, y			
<25	0.01 (0.00-0.01)	0.01 (0.00-0.02)	0.01 (0.01-0.02)
25-34	0.17 (0.15-0.19)	0.23 (0.21-0.25)	0.22 (0.21-0.23)
35-44	0.26 (0.24-0.28)	0.29 (0.26-0.31)	0.25 (0.24-0.26)
45-54	0.32 (0.30-0.34)	0.27 (0.25-0.30)	0.27 (0.26-0.28)
55-64	0.24 (0.22-0.27)	0.20 (0.18-0.23)	0.24 (0.23-0.25)
Educational level			
Less than HSG	0.01 (0.01-0.01)	0.01 (0.00-0.02)	0.04 (0.04-0.05)
HSG or GED	0.13 (0.12-0.15)	0.16 (0.13-0.18)	0.25 (0.24-0.26)
Some college or AD	0.23 (0.21-0.25)	0.22 (0.20-0.24)	0.28 (0.27-0.29)
BA or higher	0.63 (0.60-0.65)	0.61 (0.58-0.65)	0.42 (0.40-0.44)
Race and ethnicity			
Asian	0.07 (0.06-0.09)	0.06 (0.05-0.08)	0.06 (0.05-0.07)
Black	0.10 (0.08-0.11)	0.05 (0.04-0.06)	0.12 (0.11-0.13)
Hispanic	0.07 (0.06-0.08)	0.05 (0.04-0.06)	0.13 (0.12-0.15)
White	0.74 (0.72-0.76)	0.81 (0.79-0.83)	0.65 (0.64-0.67)
Multiracial or other^b^	0.02 (0.01-0.03)	0.02 (0.01-0.03)	0.03 (0.02-0.03)
Employment status			
Unemployed full year	0.00 (0.00-0.00)	0.01 (0.00-0.01)	0.02 (0.02-0.02)
Unemployed partial year	0.02 (0.01-0.02)	0.02 (0.01-0.03)	0.03 (0.02-0.03)
Employed full year	0.98 (0.98-0.99)	0.97 (0.96-0.98)	0.95 (0.95-0.96)
Total marginal income tax rate, %			
<15	0.05 (0.04-0.06)	0.06 (0.05-0.07)	0.09 (0.08-0.09)
15-19	0.19 (0.17-0.21)	0.21 (0.19-0.24)	0.29 (0.28-0.30)
20-24	0.14 (0.13-0.16)	0.17 (0.15-0.19)	0.17 (0.17-0.18)
25-29	0.27 (0.25-0.29)	0.26 (0.23-0.28)	0.19 (0.18-0.20)
≥30	0.35 (0.32-0.37)	0.30 (0.27-0.33)	0.25 (0.24-0.27)

Abbreviations: AD, associate degree; BA, bachelor’s degree; ESI, employer-sponsored insurance; FSAs, flexible spending accounts; GED, general educational development; HSAs, health savings accounts; HSG, high school graduate.

^a^ Analysis of the study population using the Medical Expenditure Panel Survey data, 2011 to 2019. Income was inflation adjusted to 2023 US dollars. The sample is restricted to families with at least 1 ESI holder, surveyed for 2 years, with no member 65 years or older. Unless otherwise indicated, data are expressed as weighted proportions (95% CI).

^b^ Includes American Indian or Alaska Native and Native Hawaiian or other Pacific Islander.

### Health Care Expenditures

**Figure 1.**
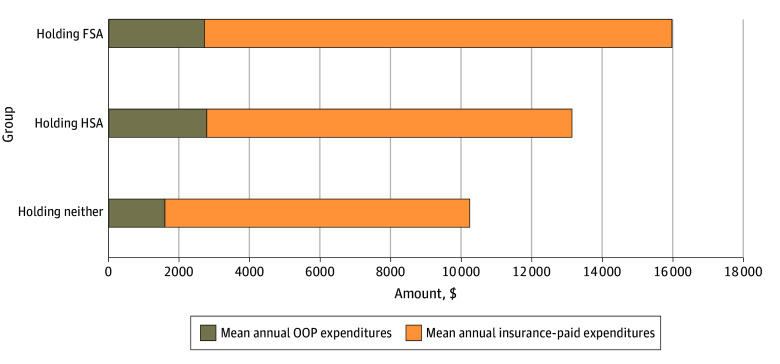
Association of a Flexible Spending Account (FSA) or a Health Savings Account (HSA) With Higher Health Care Expenditures Among Families With Employer-Sponsored Health Insurance (ESI) Analysis uses the 2011-2019 data from the Medical Expenditure Panel Survey. Year fixed-effect adjusted mean annual health care expenditures (inflation-adjusted to 2023 US dollars) were calculated from 2012 to 2019 from families with at least 1 ESI holder, surveyed for 2 years, and with no member 65 years or older. OOP indicates out-of-pocket.

In unadjusted analyses, families holding FSAs had the highest total mean annual health care expenditures ($15 987 [95% CI, $14 519-$17 455]), followed by families holding HSAs ($13 147 [95% CI, $11 490-$14 804]) and those without either tax-favored account ($10 248 [95% CI, $9705-$10 790]) (Figure 1). Mean annual out-of-pocket expenditures were highest among families holding HSAs ($2782 [95% CI, $2562-$3003]).

**Figure 2.**
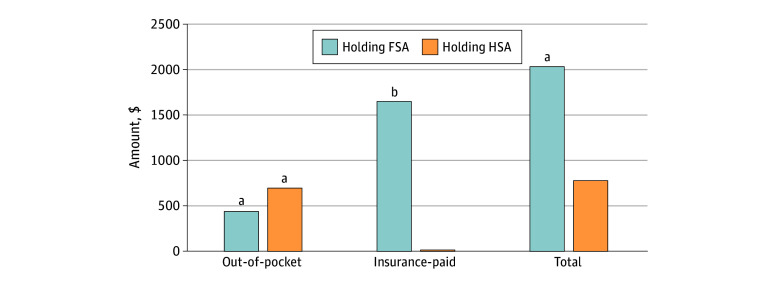
Association of Holding a Flexible Spending Account (FSA) or a Health Savings Account (HSA) With Higher Health Care Expenditures With Health Care Expenditures Relative to Families Without Accounts Analysis uses the 2011-2019 data from the Medical Expenditure Panel Survey to estimate the association of holding an FSA or HSA with annual health care expenditures from 2012 to 2019 (inflation-adjusted to 2023 US dollars). The sample is restricted to families with at least 1 holder of employer-sponsored health insurance (ESI), surveyed for 2 years, and with no member 65 years or older. The 2-part regression model controlled for family size, income, region, number of ESI beneficiaries, number of members 45 years or older, number of members with chronic conditions, and policyholder’s age, sex, race or ethnicity, educational attainment, employment, and marital status in the current year and health care expenditures and marginal tax rates in the prior year. ^a^*P* < .01. ^b^*P* < .05.

In regression-adjusted analyses, holding an FSA is associated with a mean increase of health care spending of $2033 (95% CI, $789-$3276; *P* = .001), or 20%, relative to not holding either type of account (Figure 2) and with a mean increase of $1650 (95% CI, $452-$2848; *P* = .007) or 20% higher insurer-paid expenses relative to those without accounts. Holding an HSA is associated with a mean increase of $697 (95% CI, $521-$873; *P* < .001), or 44%, in out-of-pocket medical expenses relative to families without savings accounts. However, the insurance-paid expenditures of families holding an HSA are not different from those of families without accounts. Combined mean expenditures were insignificantly higher among HSA holders ($779 [95% CI, −$660 to $2218]) than among those without accounts (eTable 1 in Supplement 1).

### Health-Related Tax Expenditures

**Figure 3.**
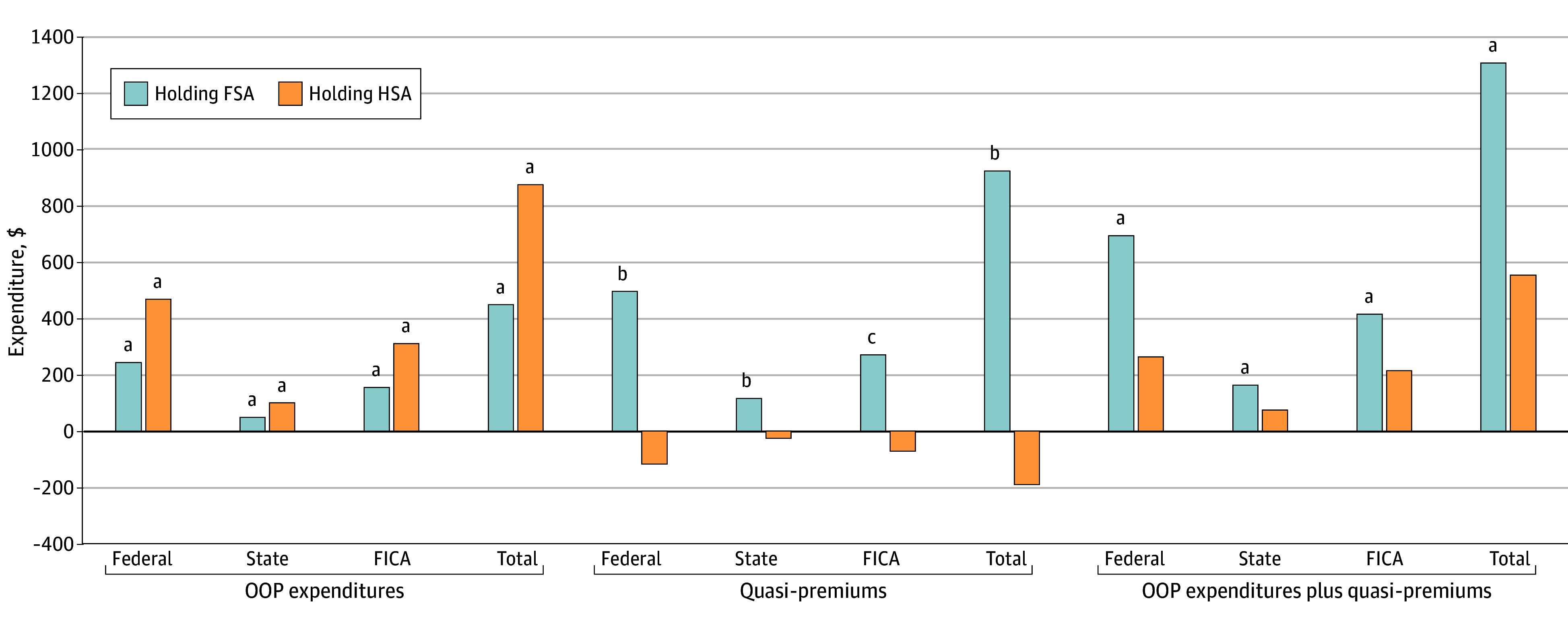
Association of Holding a Flexible Spending Account (FSA) or a Health Savings Account (HSA) With Health-Related Tax Expenditures Relative to Families Without Accounts Analysis uses the 2011-2019 data from the Medical Expenditure Panel Survey and the National Bureau of Economic Research TAXSIM model to estimate the association of holding an FSA or HSA with annual health-related tax expenditures from 2012 to 2019 (inflation-adjusted to 2023 US dollars). Note that there are no tax expenditures associated with out-of-pocket (OOP) spending among families without FSA or HSA. The sample is restricted to families with at least 1 holder of employer-sponsored health insurance (ESI) holder, surveyed for 2 years, and with no member 65 years or older. The 2-part regression model controlled for family size, income, region, number of ESI beneficiaries, number of members 45 years or older, number of members with chronic conditions, and policyholder’s age, sex, race and ethnicity, educational attainment, employment, and marital status in the current year, and health care expenditures and marginal tax rates in the prior year. FICA indicates Federal Insurance Contributions Act. ^a^*P* < .01. ^b^*P* < .05. ^c^*P* < .10.

Overall, total health-related tax expenditures of HSA holders were comparable to (but not lower than) those of similar people without savings accounts. Holding an FSA was associated with significantly higher health-related tax expenditures relative to those without accounts (Figure 3), comprised of a mean $693 (95% CI, $295-$1092; *P* = .001) greater federal income tax expenditure, $164 (95% CI, $63-$264; *P* = .001) greater state income tax expenditure, and $415 (95% CI, $121-$710; *P* = .006) greater payroll tax expenditures (FICA). Holding an HSA was associated with a mean of $875 (95% CI, $831-$918; *P* < .001) in additional tax expenditures from higher out-of-pocket spending and no significant reduction in tax expenditures through premium savings.

**Figure 4.**
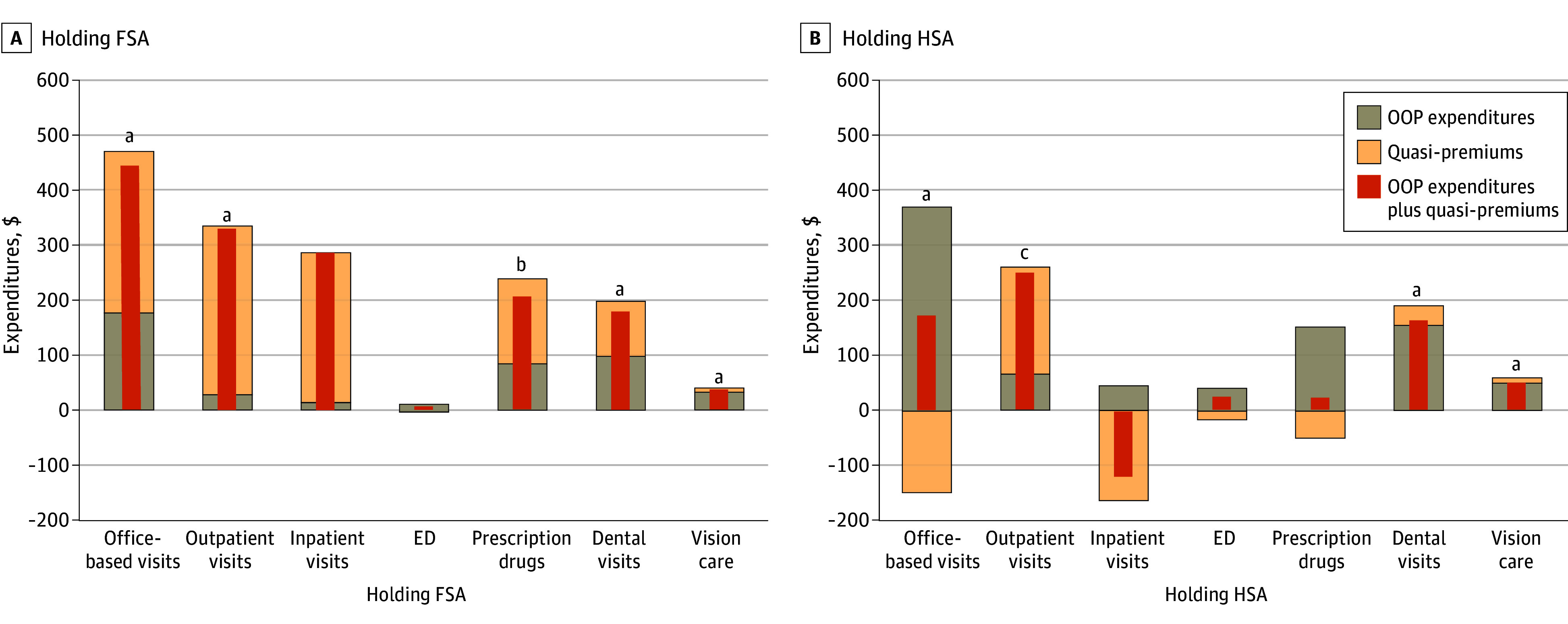
Association of Holding a Flexible Spending Account (FSA) or a Health Savings Account (HSA) With Health-Related Tax Expenditures by Service Type Relative to Families Without Accounts Analysis used 2011-2019 data from the Medical Expenditure Panel Survey and the National Bureau of Economic Research TAXSIM model to estimate the association of holding an FSA or /HSA with annual health-related tax expenditures by services from 2012 to 2019 (inflation-adjusted to 2023 US dollars). The sample is restricted to families with at least 1 holder of employer-sponsored health insurance (ESI), surveyed for 2 years, and with no member 65 years or older. The 2-part regression model controlled for family size, income, region, number of ESI beneficiaries, number of members 45 years or older, number of members with chronic conditions, and policyholder’s age, sex, race and ethnicity, educational attainment, employment, marital status in the current year, and health care expenditures and marginal tax rates in the prior year. ED indicates emergency department; OOP, out-of-pocket. ^a^*P* < .01. ^b^*P* < .10. ^c^*P* < .05.

Holding an FSA was associated with greater tax expenditures (mean of $1306 [95% CI, $536-$2076] annually per family) through both out-of-pocket spending and premiums for every service we examined, with the greatest increases in office-based ($444 [95% CI, $224-$645]; *P* < .001]) and outpatient ($330 [95% CI, $132-$528]; *P* = .001]) spending (Figure 4), though effects on inpatient and emergency department care were not statistically significant. Holding an HSA was associated with greater tax expenditures through higher out-of-pocket spending for all services we examined, but lower insurance-paid spending (and premium-associated tax expenditures) for office-based visits, inpatient stays, emergency department visits, and prescription drugs. Holding an FSA was associated with greater tax expenditures through both out-of-pocket spending and premiums for every service we examined, with the greatest increases in office-based visits ($370 [95% CI, $344-$395]; *P* < .001]), outpatient visits ($67 [95% CI, $55-$79]; *P* < .001]), dental visits ($156 [95% CI, $135-$177]; *P* < .001]), and vision care ($50 [95% CI, $44-$57]; *P* < .001]). There were no services for which HSA holders had significantly lower total spending.

### Robustness Tests

We repeated our analyses using ordinary least squares regression with top-coded dependent variables^33^ (eTable 2 in Supplement 1). In this specification, a positive association was apparent between holding HSAs and total tax expenditures. No such association was observed in the main model. We also repeated the analysis with natural logarithm-transformed dependent variables. In this specification, positive associations between account holding and health-related tax expenditures were apparent for both FSAs (0.587 [95% CI, 0.470-0.704]; *P* < .001]) and HSAs (0.592 [95% CI, 0.477-0.706]; *P* < .001]). In our main analyses, we also did not control for holding an HDHP because we were interested in the combined association of HSAs on expenditures through out-of-pocket spending and premiums, including any HDHP enrollment effect. When we controlled for HDHP status, we found that enrollment in an HDHP was not associated with overall and insurance-paid spending and overall tax expenditures. A positive association of holding an HSA with tax expenditures became apparent after controlling for HDHP status (eTable 3 in Supplement- 1). In sum, our preferred specification estimates were more conservative than plausible alternatives.

Other unobserved factors, such as an anticipated change in future health status, may be associated with decisions to hold an FSA or an HSA, with amounts contributed to such accounts and with health spending. To assess the likely importance of such factors, we repeated our analysis including an additional measure of health status (the number of family members by self-perceived health status; many observations for this variable were missing so we did not include it in our preferred specification). We found while health status measures were associated with health care expenditures and tax expenditures, their inclusion was not associated with the holding FSA or HSA coefficients. Because we imputed contribution amounts for HSAs, the choice of contribution level did not bias our HSA analysis. We repeated our analysis similarly imputing contribution amounts for FSAs. Our FSA results were not sensitive to this change (see eTable 4 in Supplement 1). Together, these tests suggested that omitted variable bias was unlikely to be an important explanation of our results.

To further test the potential magnitude of unobserved variable bias, we compared the coefficients on holding FSA and holding HSA in models with and without observable controls and calculated the degree of proportionality (δ) between observed and potential unobserved variables, as suggested by Oster.^34^ Under Oster’s recommended assumption, we calculated the bias-adjusted effects of holding FSAs and holding HSAs associated with each health care expenditure variable (eTable 5 in Supplement 1) and concluded that the estimates from our main model were likely to be robust to plausible levels of unobserved variable bias.

## Discussion

Proponents of tax-favored savings accounts assert that these accounts can offset the distortionary effects generated by the favorable tax treatment of ESI by reducing the incentive to insure expenses.^11,12,13^ We find that this is not the case in this cross-sectional study. Both FSAs and HSAs reduce the after-tax cost of out-of-pocket expenditures and, as expected, lower out-of-pocket costs are associated with higher out-of-pocket spending. For FSA holders, however, account holding is also associated with significant and sizable increases in insurance-paid expenditures, overall and for each specific service we examine. Lower out-of-pocket cost-sharing is associated with increased use of health services, not health expenditure savings.

Health savings accounts were designed to avoid this outcome. They must be coupled with HDHPs and were intended to encourage enrollment in these cost-reducing plans.^11,35^ Our results, consistent with prior evidence, suggest that the hoped-for result of lower spending does not occur.^7^ Holding an HSA is associated with higher tax-favored out-of-pocket spending, but despite the requirement that HSA holders enroll in HDHPs, it is not associated with lower insurance-paid spending. Holding an HSA is associated with significantly higher tax expenditures for outpatient visits, office-based visits, dental care, and vision care and total estimated tax expenditures that are insignificantly higher than those of non–account holders.

Policymakers understandably like policies that are popular among constituents, seem to be costless, and are posited to improve efficiency. Tax-favored accounts are certainly popular, as they offer holders, especially those with higher incomes, a substantial discount on cost-sharing and services such as dental and vision care that are not always covered by insurance. Because tax-favored accounts do not constitute direct government spending, legislators may see them as costless. Advocates have argued that these accounts may improve the efficiency of the system and reduce expenditures by drawing people into plans with higher deductibles and cost-sharing.

Unfortunately, there are no free lunches in the health care system. The additional tax-favored spending generated by account holders is a benefit to them, but our results suggest that it does not increase and may reduce the efficiency of the health care system. Policymakers should be cautious about expanding such accounts further.

### Strengths and Limitations

Our findings provide new evidence for understanding the association between holding FSAs or HSAs and health care expenditures, using data since 2011, a period in which deductibles have increased across the health care sector.^36^ However, our analysis has several limitations. First, MEPS does not report contributions to HSAs, and we estimated these based on a survey. Second, public-use MEPS data do not include state identifiers, and we randomly assigned families to states within US Census regions. Third, we computed quasi-premiums by calibrating the household survey to insurer data, but this calibration likely varies by year (due to changes in the MEPS survey^37^), and we were not able to adjust for such changes. Fourth, we may underestimate total tax expenditures by excluding eligible over-the-counter spending (not collected in MEPS) and account-holding families who are no longer enrolled in ESI but continue using their funds.

## Conclusions

Findings of this cross-sectional study suggest that the availability of FSAs is associated with higher health care expenditures and the availability of HSAs is not associated with lower health care expenditures, consistent with the hypothesis that these vehicles undermine cost-sharing incentives in health plans. Because these plans are more valuable to and more popular among high-income, highly educated White households, future research should explore how their availability affects inequities in the use of health services. Tax policy could be better targeted to enhance insurance coverage and health care accessibility.

## Notes

Supplement 1. **eTable 1.** Coefficients on Holding FSA or HSA From a Two-Part Model **eTable 2.** Association of Holding FSA or HSA on Health Care Expenditure and Health-Related Tax Expenditures Using Different Models **eTable 3.** Association of Holding FSA or HSA on Health-Related Tax Expenditures Controlling for High-Deductible Health Plan Status **eTable 4.** Association of Holding FSA or HSA on Health-Related Tax Expenditures Using Imputed FSA Contribution **eTable 5.** Results From Oster Test

Supplement 2.
Data Sharing Statement
