# Supplementary material for: Health Care–Related Savings Accounts, Health Care Expenditures, and Tax Expenditures
Source: JAMA Health Forum. 2024 Sep 20;5(9):e242896. doi: 10.1001/jamahealthforum.2024.2896 (PMC11415789; doi:10.1001/jamahealthforum.2024.2896)
Supplement: Supplement 1. — eTable 1. Coefficients on Holding FSA or HSA From a Two-Part Model eTable 2. Association of Holding FSA or HSA on Health Care Expenditure and Health-Related Tax Expenditures Using Different Models eTable 3. Association of Holding FSA or HSA on Health-Related Tax Expenditures Controlling for High-Deductible Health Plan Status eTable 4. Association of Holding FSA or HSA on Health-Related Tax Expenditures Using Imputed FSA Contribution eTable 5. Results From Oster Test [file jamahealthforum-e242896-s001.pdf]

Supplementary Online Content

Ding D, Glied S. Health care–related savings accounts, health care expenditures, and tax expenditures. *JAMA Health Forum*. 2024;5(9):e242896. doi:10.1001/jamahealthforum.2024.2896

eTable 1. Coefficients on Holding FSA or HSA From a Two-Part Model

eTable 2. Association of Holding FSA or HSA on Health Care Expenditure and Health-Related Tax Expenditures Using Different Models

eTable 3. Association of Holding FSA or HSA on Health-Related Tax Expenditures Controlling for High-Deductible Health Plan Status

eTable 4. Association of Holding FSA or HSA on Health-Related Tax Expenditures Using Imputed FSA Contribution

eTable 5. Results From Oster Test

This supplementary material has been provided by the authors to give readers additional information about their work.

© 2024 Ding D et al. *JAMA Health Forum*.

eTable 1. Coefficients on Holding FSA or HSA From a Two-Part Model

		Healthcare Expenditures			Quasi-premiums	Tax Expenditures on Pre-tax OOP Expenditures				Tax Expenditures on Quasi-premiums				Tax Expenditures on OOP Expenditure[illegible] Quasi-premiums			
		**(1)**	**(2)**	**(3)**	**(4)**	**(5)**	**(6)**	**(7)**	**(8)**	**(9)**	**(10)**	**(11)**	**(12)**	**(13)**	**(14)**	**(15)**	[illegible]
		Total	***OOP***	***Insurance-paid***	***Quasi-premiums***	***Federal***	***State***	***FICA***	***Total***	***Federal***	***State***	***FICA***	***Total***	***Federal***	***State***	***FICA***	[illegible]
**Probit**	**Holding FSA**	0.118	0.100	0.236**	0.132**	reference	reference	reference	reference	0.137**	-0.016	0.132**	0.134**	0.361***	0.048	0.402***	[illegible]
		(0.091)	(0.073)	(0.075)	(0.056)					(0.051)	(0.042)	(0.056)	(0.056)	(0.062)	(0.044)	(0.072)	[illegible]
	**Holding HSA**	0.237**	0.128*	0.115	0.064	0.764***	0.393***	0.851***	0.851***	0.069	0.032	0.064	0.065	0.505***	0.170***	0.595***	[illegible]
		(0.095)	(0.072)	(0.075)	(0.063)	(0.067)	(0.051)	(0.073)	(0.073)	(0.058)	(0.046)	(0.063)	(0.063)	(0.067)	(0.045)	(0.083)	[illegible]
**GLM**	**Holding FSA**	0.167**	0.221***	0.152**	0.150**	reference	reference	reference	reference	0.158**	0.179**	0.115*	0.151**	0.201**	0.222**	0.159**	[illegible]
		(0.050)	(0.036)	(0.057)	(0.061)					(0.063)	(0.068)	(0.064)	(0.063)	(0.061)	(0.066)	(0.062)	[illegible]
	**Holding HSA**	0.062	0.331***	-0.005	-0.021	0.554***	0.566***	0.587***	0.565***	-0.052	-0.052	-0.042	-0.043	0.055	0.061	0.064	[illegible]
		(0.063)	(0.039)	(0.075)	(0.081)	(0.035)	(0.385)	(0.037)	(0.034)	(0.079)	(0.094)	(0.083)	(0.080)	(0.072)	(0.086)	(0.075)	[illegible]
**Overall**	**Holding FSA**	2,033.7***	439.4***	1,650.3***	2,433.2**	reference	reference	reference	reference	495.7**	116.3**	271.4*	924.0**	693.3***	163.7***	415.3***	[illegible]
		(632.3)	(75.8)	(609.2)	(998.6)					(198.3)	(50.2)	(146.7)	(383.7)	(202.6)	(51.1)	(149.7)	[illegible]
	**Holding HSA**	779.3	697.0***	15.42	-207.2	224.9***	50.9***	155.3***	425.7***	-116.4	-24.7	-70.8	-189.8	264.2	74.8	214.9	[illegible]
		(731.9)	(89.7)	(694.2)	(1,111.5)	(13.8)	(3.4)	(9.5)	(24.7)	(203.0)	(56.9)	(160.7)	(404.8)	(207.0)	(58.0)	(164.0)	[illegible]
**N**		17,038	17,038	17,038	17,038	4,473	4,473	4,452	4,452	17,038	17,038	17,038	17,038	17,038	17,038	17,038	[illegible]

Standard errors are in parentheses. *** p<0.01, ** p<0.05, * p<0.1.
Note: Author’s analysis using the Medical Expenditure Panel Survey data, 2011-2019, and the NBER TAXSIM model. Expenditures are inflation-adjusted to 2023 US dollars. The sample is restricted to families with at least one ESI holder, surveyed for two years, with no member 65 years or older. The two-part regression model controlled for family size, income, region, number of ESI beneficiaries, number of members 45 years or older, number of members with chronic conditions, and policyholder’s age, sex, race or ethnicity, educational attainment, employment, and marital status in the current year, and healthcare expenditures and marginal tax rates in the prior year. Note that there are no tax expenditures associated with out-of-pocket spending among families without FSA/HSA accounts. Abbreviations: OOP, out-of-pocket; FSA, flexible spending account; HSA, health savings account; ESI, employer-sponsored insurance; FICA, Federal Insurance Contributions Act.

© 2024 Ding D et al. *JAMA Health Forum*.

eTable 2. Association of Holding FSA or HSA on Health Care Expenditure and Health-Related Tax Expenditures Using Different Models

	Healthcare Expenditures			Quasi-premiums	Tax Expenditures on Pre-tax OOP Expenditures				Tax Expenditures on Quasi-premiums				Tax Expenditures on OOP Expenditures + Quasi-premiums			
	(1)	(2)	(3)	(4)	(5)	(6)	(7)	(8)	(9)	(10)	(11)	(12)	(13)	(14)	(15)	(16)
	Total	*OOP*	*Insurance-paid*	*Quasi-premiums*	*Federal*	*State*	*FICA*	*Total*	*Federal*	*State*	*FICA*	*Total*	*Federal*	*State*	*FICA*	*Total*
**Two-part Model**																
**Holding FSA**	2,033.7***	439.4***	1,650.3***	2,433.2**	243.9***	49.5***	156.1***	448.8***	495.7**	116.3**	271.4*	924.0**	693.3***	163.7***	415.3***	1,305.9***
	(632.3)	(75.8)	(609.2)	(998.6)	(6.2)	(1.5)	(4.3)	(11.0)	(198.3)	(50.2)	(146.7)	(383.7)	(202.6)	(51.1)	(149.7)	(391.6)
**Holding HSA**	779.3	697.0***	15.42	-207.2	468.8***	100.4***	311.4***	874.6***	-116.4	-24.7	-70.8	-189.8	264.2	74.8	214.9	553.9
	(731.9)	(89.7)	(694.2)	(1,111.5)	(12.7)	(3.3)	(8.5)	(22.2)	(203.0)	(56.9)	(160.7)	(404.8)	(207.0)	(58.0)	(164.0)	(411.7)
**N**	17,038	17,038	17,038	17,038	4,473	4,473	4,452	4,452	17,038	17,038	17,038	17,038	17,038	17,038	17,038	17,038
**Top-coded OLS**																
**Holding FSA**	2,077.2***	448.0***	1,593.2***	2,241.3***	234.6***	47.80***	153.5***	437.8***	510.0***	105.4**	222.7*	872.6***	724.8***	150.9***	369.8***	1,280.7***
	(556.8)	(68.5)	(515.0)	(836.9)	(5.8)	(1.5)	(4.0)	(10.5)	(165.6)	(43.8)	(121.9)	(315.7)	(166.0)	(44.3)	(123.6)	(317.4)
**Holding HSA**	593.5	737.5***	-188.3	-456.7	425.9***	89.79***	292.7***	815.7***	-88.9	-18.3	-81.7	-215.0	322.8*	70.82	205.7*	572.4*
	(543.8)	(93.3)	(498.0)	(811.7)	(9.5)	(2.5)	(6.9)	(17.3)	(165.1)	(44.2)	(119.5)	(307.7)	(167.7)	(45.0)	(120.6)	(310.5)
**R-squared**	0.17	0.20	0.15	0.14	0.33	0.24	0.28	0.30	0.15	0.12	0.13	0.14	0.16	0.12	0.14	0.15
**N**	17,038	17,038	17,038	17,038	4,473	4,473	4,473	4,473	17,038	17,038	17,038	17,038	17,038	17,038	17,038	17,038
**ln transformed OLS**																
**Holding FSA**	0.269***	0.340***	0.289***	0.269***	4.332***	2.560***	4.006***	4.917***	0.284***	0.155**	0.221***	0.267***	0.585***	0.359***	0.518***	0.587***
	(0.048)	(0.048)	(0.051)	(0.065)	(0.048)	(0.049)	(0.047)	(0.053)	(0.061)	(0.078)	(0.061)	(0.062)	(0.058)	(0.078)	(0.059)	(0.060)
**Holding HSA**	0.182***	0.479***	0.011	-0.044	5.322***	3.311***	5.008***	6.017***	-0.056	-0.044	-0.065	-0.052	0.549***	0.388***	0.529***	0.592***
	(0.054)	(0.057)	(0.062)	(0.073)	(0.047)	(0.058)	(0.043)	(0.047)	(0.067)	(0.080)	(0.063)	(0.068)	(0.059)	(0.076)	(0.055)	(0.058)
**R-squared**	0.43	0.40	0.40	0.38	0.26	0.25	0.26	0.25	0.38	0.27	0.38	0.39	0.40	0.28	0.40	0.41
**N**	17,038	17,038	17,038	17,038	4,473	4,473	4,473	4,473	17,038	17,038	17,038	17,038	17,038	17,038	17,038	17,038

Standard errors are in parentheses. *** p<0.01, ** p<0.05, * p<0.1.

Note: Author's analysis using the Medical Expenditure Panel Survey data, 2011-2019, and the NBER TAXSIM model. Average marginal effects of holding FSA/HSA on annual healthcare expenditures and tax expenditures from 2012 to 2019 (inflation-adjusted to 2023 US dollars). The sample is restricted to families with at least one ESI holder, surveyed for two years, with no member 65 years or older. Note that there are no tax expenditures associated with out-of-pocket spending among families without FSA/HSA accounts. All models controlled for family size, income, region, number of ESI beneficiaries, number of members 45 years or older, number of members with chronic conditions, and policyholder's age, sex, race and ethnicity, educational attainment, employment, marital status, and healthcare expenditures and marginal tax rates in the prior year. Abbreviations: FSA, flexible spending account; HSA, health savings account; ESI, employer-sponsored insurance; OOP, out-of-pocket; FICA, Federal Insurance Contributions Act.

© 2024 Ding D et al. *JAMA Health Forum*.

eTable 3. Association of Holding FSA or HSA on Health-Related Tax Expenditures Controlling for High-Deductible Health Plan Status

	Healthcare Expenditures			Quasi-premiums	Tax Expenditures on Pre-tax OOP Expenditures				Tax Expenditures on Quasi-premiums				Tax Expenditures on OOP Expenditures + Quasi-premiums			
	(1)	(2)	(3)	(4)	(5)	(6)	(7)	(8)	(9)	(10)	(11)	(12)	(13)	(14)	(15)	(16)
	Total	*OOP*	*Insurance-paid*	*Quasi-premiums*	*Federal*	*State*	*FICA*	*Total*	*Federal*	*State*	*FICA*	*Total*	*Federal*	*State*	*FICA*	*Total*
**HDHP**	-378.7	296.2***	-676.7	-970.3	78.14***	23.0***	56.1***	153.8***	-275.4*	-38.2	-131.0	-432.8	-268.7	-34.25	-120.9	-412.4
	(574.8)	(79.6)	(529.5)	(851.1)	(14.2)	(3.7)	(9.6)	(25.9)	(162.3)	(43.4)	(123.5)	(317.4)	(167.9)	(44.5)	(127.0)	(327.3)
**Holding FSA**	2,044***	439.1***	1,675***	2,466**	263.5***	55.0***	170.5***	488.0***	504.1**	117.0**	275.2*	937.5**	701.7***	164.4***	418.9***	1,319***
	(627.9)	(76.98)	(602.2)	(988.5)	(7.1)	(1.9)	(4.9)	(12.7)	(195.5)	(49.8)	(145.5)	(379.3)	(199.7)	(50.7)	(148.5)	(387.2)
**Holding HSA**	1,079	447.3***	537.2	531.4	429.0***	88.14***	283.4***	797.5***	92.6	3.9	27.3	137.2	489.0*	103.3	314.2	893.6*
	(865.7)	(108.1)	(820.6)	(1,311)	(13.8)	(3.454)	(9.2)	(24.5)	(244.1)	(65.2)	(190.3)	(480.0)	(254.9)	(67.8)	(198.4)	(499.0)
**N**	17,038	17,038	17,038	17,038	4,473	4,473	4,452	4,452	17,038	17,038	17,038	17,038	17,038	17,038	17,038	17,038

Standard errors are in parentheses. *** p<0.01, ** p<0.05, * p<0.1.

Note: Author's analysis using the Medical Expenditure Panel Survey data, 2011-2019, and the NBER TAXSIM model. Expenditures are inflation-adjusted to 2023 US dollars. The sample is restricted to families with at least one ESI holder, surveyed for two years, with no member 65 years or older. The two-part regression model controlled for family size, income, region, number of ESI beneficiaries, number of members 45 years or older, number of members with chronic conditions, and policyholder's age, sex, race and ethnicity, educational attainment, employment, HDHP, and marital status in the current year, and healthcare expenditures and marginal tax rates in the prior year. Note that there are no tax expenditures associated with out-of-pocket spending among families without FSA/HSA accounts. Abbreviations: HDHP, high-deductible health plan; OOP, out-of-pocket; FSA, flexible spending account; HSA, health savings account; ESI, employer-sponsored insurance; FICA, Federal Insurance Contributions Act.

© 2024 Ding D et al. *JAMA Health Forum*.

eTable 4. Association of Holding FSA or HSA on Health-Related Tax Expenditures Using Imputed FSA Contribution

	Tax Expenditures on Pre-tax OOP Expenditures			
	**(1)**	**(2)**	**(3)**	**(4)**
	Federal	***State***	***FICA***	***Total***
**Holding FSA**	264.3***	55.7***	172.7***	491.6***
	(4.4)	(1.3)	(3.2)	(7.8)
**Holding HSA**	459.5***	97.5***	307.7***	860.2***
	(12.2)	(3.2)	(8.2)	(21.4)
**N**	4,473	4,473	4,452	4,452

Standard errors are in parentheses. *** p<0.01, ** p<0.05, * p<0.1.
Note: Author's analysis using the Medical Expenditure Panel Survey data, 2011-2019, and the NBER TAXSIM model. Average marginal effects of holding an FSA/HSA on annual health-related tax expenditures from 2012 to 2019 (inflation-adjusted to 2023 US dollars). The FSA-holding family's tax-sheltered out-of-pocket expenditure threshold is defined as the average FSA contribution by age group. The sample is restricted to families with at least one ESI holder, surveyed for two years, with no member 65 years or older. Note that there are no tax expenditures associated with out-of-pocket spending among families without FSA/HSA accounts. All models controlled for family size, income, region, number of ESI beneficiaries, number of members 45 years or older, number of members with chronic conditions, and policyholder's age, sex, race and ethnicity, educational attainment, employment, marital status, and healthcare expenditures and marginal tax rates in the prior year. Abbreviations: FSA, flexible spending account; HSA, health savings account; ESI, employer-sponsored insurance; OOP, out-of-pocket; FICA, Federal Insurance Contributions Act.

© 2024 Ding D et al. *JAMA Health Forum*.

eTable 5. Results From Oster Test

Using formula from Oster (Oster 2019, p193): $\beta^* \approx \tilde{\beta} - \delta[\dot{\beta} - \tilde{\beta}]\frac{R_{max}-\tilde{R}}{\tilde{R}-\dot{R}}$,

where $\dot{\beta}$ and $\dot{R}$ represent the coefficient and the R^2 from the models without controls; $\tilde{\beta}$ and $\tilde{R}$ represent the coefficient and R^2 from the models with controls. Under the assumption of R_{max}=1.3$\tilde{R}$ suggested by Oster, we calculated β^*, the bias-adjusted effects, of holding FSAs or HSAs associated with each healthcare expenditures variable.

		Total Expenditures	OOP Expenditures	Insurance-paid Expenditures
$\tilde{R}$		0.173	0.202	0.147
R_{max}		0.225	0.262	0.191
Holding FSA	$\dot{R}$	0.010	0.014	0.008
	δ	0.134	0.242	0.101
	$\dot{\beta}$	4723	812	3847
	β^*	***1953***	***417***	***1514***
	$\tilde{\beta}$	**2077**	**448**	**1593**
Holding HSA	$\dot{R}$	0.001	0.017	0.000
	δ	0.086	0.509	-0.030
	$\dot{\beta}$	1861	988	816
	β^*	***558***	***691***	***-178***
	$\tilde{\beta}$	**594**	**737**	**-188**

Note: Abbreviations: FSA, flexible spending account; HSA, health savings account; OOP, out-of-pocket.

© 2024 Ding D et al. *JAMA Health Forum*.
