# Supplementary material for: Health Care–Related Savings Accounts, Health Care Expenditures, and Tax Expenditures
Source: JAMA Health Forum. 2024 Sep 20;5(9):e242896. doi: 10.1001/jamahealthforum.2024.2896 (PMC11415789; doi:10.1001/jamahealthforum.2024.2896)
Supplement: Supplement 2. — Data Sharing Statement [file jamahealthforum-e242896-s002.pdf]

Data Sharing Statement

Ding. Health Care–Related Savings Accounts, Health Care Expenditures, and Tax Expenditures. *JAMA Health Forum*. Published September 20, 2024. doi:10.1001/jamahealthforum.2024.2896

Data
Data available: No

Additional Information
Explanation for why data not available: We used publicly accessible data in this survey. Data sources are provided in the reference list. We would love to provide our final analytical dataset as well as Stata code to replicate the study.
